# Uncovering early events in primary Epstein-Barr virus infection using a rabbit model

**DOI:** 10.1038/s41598-021-00668-x

**Published:** 2021-10-27

**Authors:** Narendran Reguraman, Asma Hassani, Pretty Philip, Gulfaraz Khan

**Affiliations:** Department of Microbiology and Immunology, College of Medicine and Health Sciences, Tawam Hospital Campus, United Arab Emirates University, Al Ain, United Arab Emirates

**Keywords:** Herpes virus, Viral pathogenesis

## Abstract

Epstein-Barr virus (EBV) is an oncogenic herpesvirus implicated in the pathogenesis of several malignant and non-malignant conditions. However, a number of fundamental aspects about the biology of EBV and the mechanism(s) by which this virus induces pathology remain unknown. One major obstacle has been the lack of a suitable animal model for EBV infection. In this study, using our recently established rabbit model of EBV infection, we examined the early events following primary EBV infection. We show that, both immunocompetent and immunosuppressed animals were readily susceptible to EBV infection. However, immunosuppressed animals showed marked splenomegaly and widespread infection. Following EBV infection*,* the virus primarily targeted naïve IgM^+^, CD20^+^, CD21^+^ and CD79a^+^ B cells. Infected cells expressed varying sets of viral latent/lytic gene products. Notably, co-expression of latent and lytic proteins in the same cell was not observed. Infected cells in type 0/1 latency (EBERs^+^), were small and proliferating (Ki67^+^). By contrast, cells in type 2/3 latency (LMP1^+^), were large, non-proliferating (Ki-67^−^) and p53^+^. Although infected B-cells were widely present in splenic follicles, they did not express germinal center marker, BCL-6. Taken together, this study shows for the first time, some of the early events following primary EBV infection.

## Introduction

Epstein-Barr virus (EBV) is a common herpesvirus associated with the pathogenesis of a number of malignant and non-malignant conditions. A large part of our current understanding of the biology of EBV is based on in vitro studies of infection of human B-lymphocytes. In vitro infection of B-cells leads to their immortalization^1^. In these cells, the virus establishes a latent infection, in which upto 11 viral products, namely 6 Epstein-Barr nuclear antigens (EBNA1, EBNA2, EBNA3a, EBNA3b, EBNA3c, EBNA-LP), 3 latent membrane proteins (LMP1, LMP2a, LMP2b) and large quantities of two non-protein encoding RNAs (EBER-1 and EBER-2) are expressed^2^. This is referred to as type III latency or growth program.

By contrast, the biology of EBV infection in vivo is much more complex and less well understood. Although it is known that EBV is transmitted via the oral route, it is unclear whether B-cells or oropharyngeal squamous epithelial cells are the initial cellular targets of EBV^3–5^. In healthy carrier, circulating memory B cells are believed to constitute the main virus reservoir. However, EBV can also infect T cells and NK cells in T/NK cell lymphomas, and epithelial cells in certain malignancies such as nasopharyngeal carcinoma and gastric cancer^6–8^. What is clear, is that EBV establishes a life-long persistence in resting memory B-lymphocytes^9^. In these cells, the virus expresses little or no viral proteins. This is referred to as type 0 latency^10^. However, the details of the early events in primary EBV infection in vivo remain unknown^11^. One major obstacle, which has hampered research in uncovering the early events in EBV biology, has been the lack of a suitable animal model. EBV is highly cell tropic, infecting only human B-cells expressing CD21 receptor^12^. B-cells from animals such as mice or rats cannot be infected with EBV, in vivo or in vitro. EBV-like viruses infecting mice or primates^13–17^ have been used to study EBV, as have humanized mice and Chinese tree shrews^18–20^. These models, albeit useful, have their drawbacks and do not fully recapitulate natural infection in humans^21^. Thus, without a suitable animal model, a number of fundamental questions relating to the early events in primary EBV infection remain outstanding.

Recent studies have shown that EBV can infect rabbits by different routes of administration, such as intravenous and intranasal^22, 23^. Previously, we have shown that healthy rabbits are susceptible to EBV infection and the virus establishes long-term persistence without causing any obvious pathologies^24, 25^. These features are similar to latent EBV infection observed in humans. Furthermore, immunosuppression of latently infected rabbits leads to proliferation and widespread dissemination of EBV infection, comparable to that seen in post-transplant patients on immunosuppressive therapy. It has been hypothesized that the germinal centers (GCs) of the secondary lymphoid tissues are fundamental, not only for EBV persistence, but also for proliferation of EBV infected cells^26–29^.

In this study, we used our established rabbit model to understand the early events in EBV biology and determine the cellular targets and role of GCs in primary EBV infection. Our findings show that naïve B-cells are the primary target for the virus. The infected cells appear to be a heterogeneous population, expressing different viral latency programs. Notably, the EBV infected cells proliferating in the lymphoid follicles, do not appear to express germinal center B-cell marker.

## Results

### Clinical and autopsy findings

**Figure 1:**
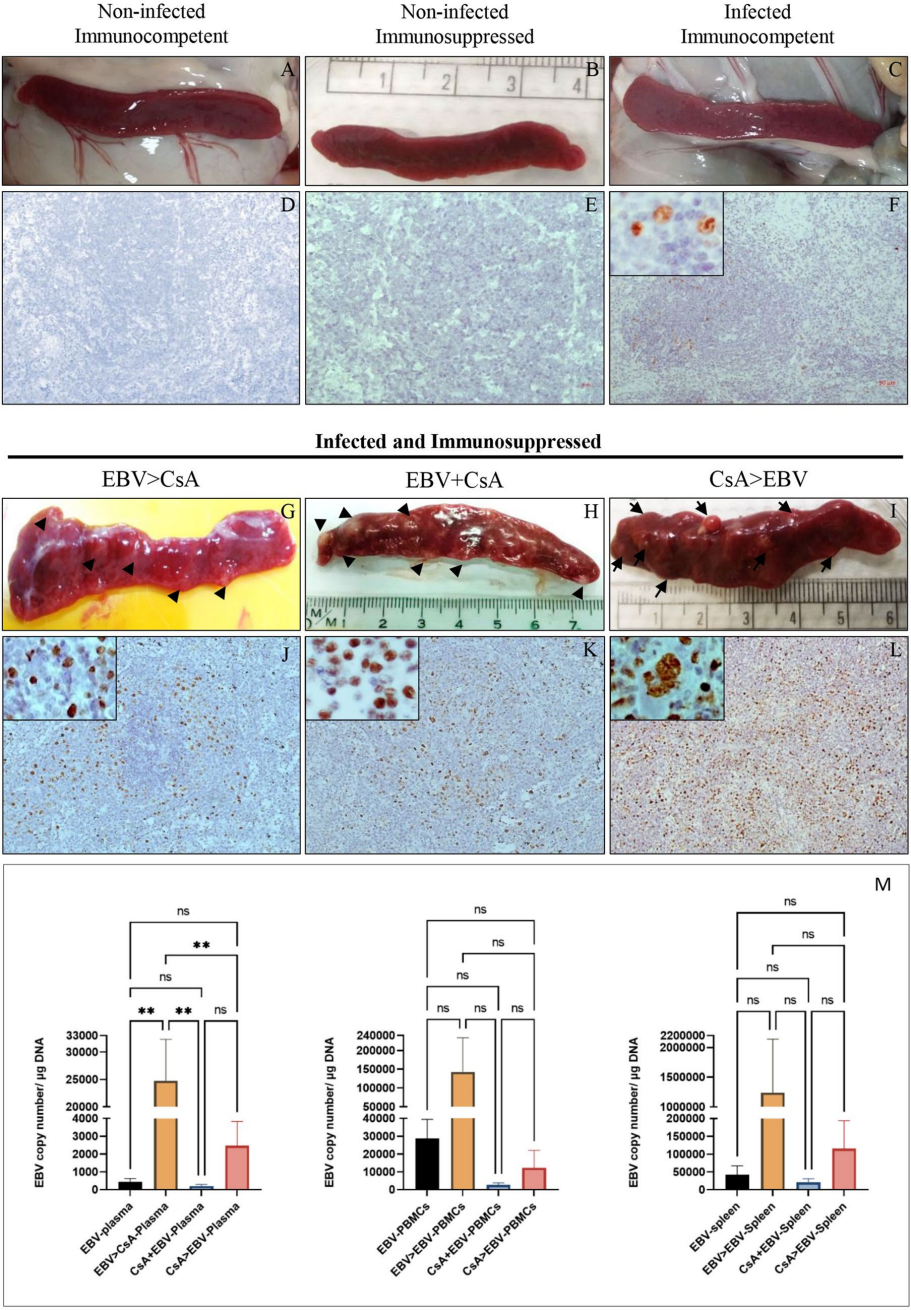
Macroscopic visualization of rabbit spleens. (**A**) Spleen of PBS and (**B**) CsA from non-infected control animals and (**C**) infected immunocompetent group. (**D–F**) EBER-ISH for the detection of EBV showed positive signals only in sections from infected animals, but not in non-infected controls. (**G**–**I**) In contrast to healthy immunocompetent animals, the spleens from infected and immunosuppressed animals were greatly enlarged and visibly abnormal. EBER-ISH indicated widespread infection in the spleen of all three immunosuppressed groups, irrespective of when immunosuppression was started, (**J**) EBV > CsA, (**K**) EBV + CsA or (**L**) CsA > EBV. (M) Comparison of viral load by qPCR indicated that the spleen had the highest load, compared to PBMCs and plasma.

Animals were monitored on daily basis by a qualified veterinarian. No clinical symptoms were observed in any of the animals in the non-infected control groups (PBS only/CsA only) or in the EBV infected immunocompetent animals (EBV only). This is consistent with our previous findings indicating that EBV infection of healthy immunocompetent rabbits results in asymptomatic long-term persistence of the virus, analogous to what has been observed in human^24, 25^. Furthermore, at autopsy, these animals also showed no gross macroscopic changes in organs such as the spleen or liver. Both of these organs were within normal size range (Fig. 1A, B). By contrast, some of the animals in the immunosuppressed groups [EBV > CsA (n = 2/6), EBV + CsA (n = 2/6) and CsA > EBV (n = 3/6)], showed signs of poor activity and weight loss during the last week of infection. Upon sacrifice, autopsy findings from these same animals revealed macroscopic changes, most notably in the spleen. The spleen was not only considerably larger (6–8 cm) than the non-infected controls (approximately 4 cm), but importantly, it was visibly abnormal, weighing 0.2–2.7 g (Fig. 1G–I). Irrespective of when immunosuppression was started (i.e. pre, same time or post EBV infection), the spleens of these animals were heavily inflamed, nodular, and in some cases had visible cysts.

### EBV is present in the spleen of infected animals

EBER in situ hybridization was used for the detection of EBV in formalin-fixed, paraffin embedded spleen sections. Although all EBV infected animals, immunocompetent and immunosuppressed, were clearly found to be infected, the degree of infection varied considerably between the two groups (Fig. 1). In the healthy immunocompetent animals, EBV infected cells were limited and scattered in the spleen (Fig. 1F). By contrast, in the immunosuppressed group, EBV infection was widespread and extensive (Fig. 1J–L), suggesting higher viral levels in infected animals with weakened immune system. Among the three immunosuppressed groups, CsA followed by EBV infection group (CsA > EBV) had the highest number of EBER^+^ cells in the spleen. Intriguingly, some of these EBER^+^ cells were markedly large and multi-nucleated, resembling Reed-Sternberg (RS) like cells of Hodgkin lymphoma (Fig. 1L). These bizarre-looking cells were primarily observed in the immunosuppressed and not in the immunocompetent animals. No EBER signal was detected in the spleen sections from the non-infected control groups (PBS or CsA) (Fig. 1D–E). We also performed qPCR to determine viral load in plasma, PBMCs and spleen from the different immunocompetent and immunosuppressed groups. Viral load in the spleen was observed to be comparatively higher than that in the plasma and PBMCs (Fig. 1M).

### EBV infected cells in the spleen express B-cell markers

To determine the phenotype of EBV infected cells in the rabbit spleen, we used double staining for different viral and cellular markers. For B-cells, we used CD19, CD20 and CD79a (all pan-B cell markers). For naïve B cells, we used IgM. Additionally, we used CD21, a B-cell marker and a known receptor for EBV. For T-cells, we used CD3 (pan-T-cell marker).

**Figure 2:**
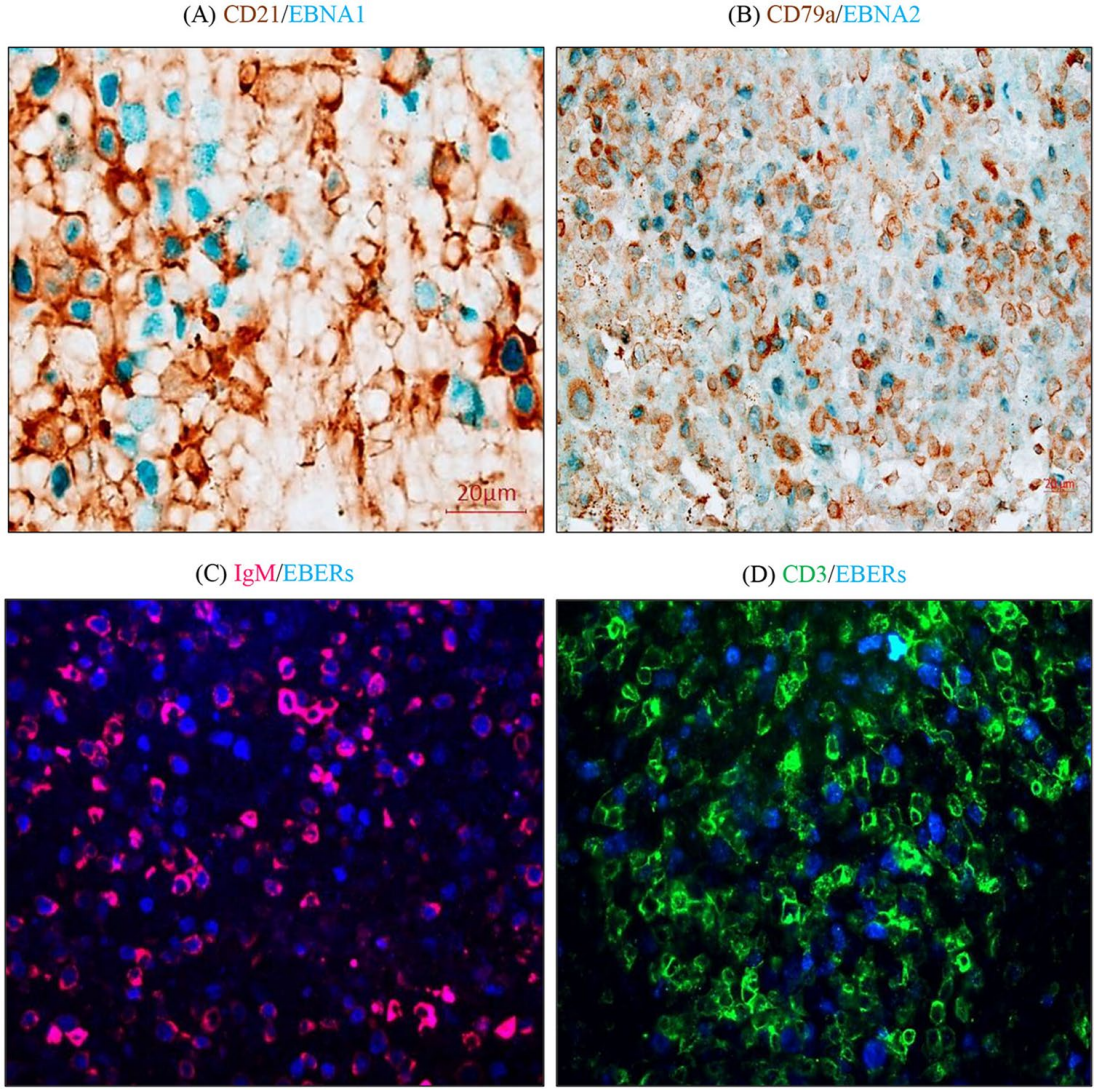
The phenotype of EBV infected cells in the spleen are B-cells. Representative spleen sections showing double-staining for EBV and B-cell markers: (**A**) CD21 (brown) and EBNA1 (blue), (**B**) CD79a (brown) and EBNA2 (blue), (**C**) IgM (pink) and EBERs (blue). (**D**) Double staining for T-cell marker CD3 (green) and EBERs (blue), indicated that T-cells were not the target of EBV infection.

Double staining clearly revealed that B-cells were the primary target for EBV. Infected cells expressed CD19, CD20, CD21 and CD79a (Fig. 2A–B and sFig. 1). A large proportion of EBV infected B-cells were found to be naïve B-cells expressing IgM (Fig. 2C and sFig. 2). Infected B cells also showed variable expression of B cell markers such as CD79a (low) and IgM (high). By contrast, we did not observe any CD3-positive T-cells to be EBV infected (Fig. 2D).

### EBV infected cells in the spleen express different viral latent proteins

**Figure 3:**
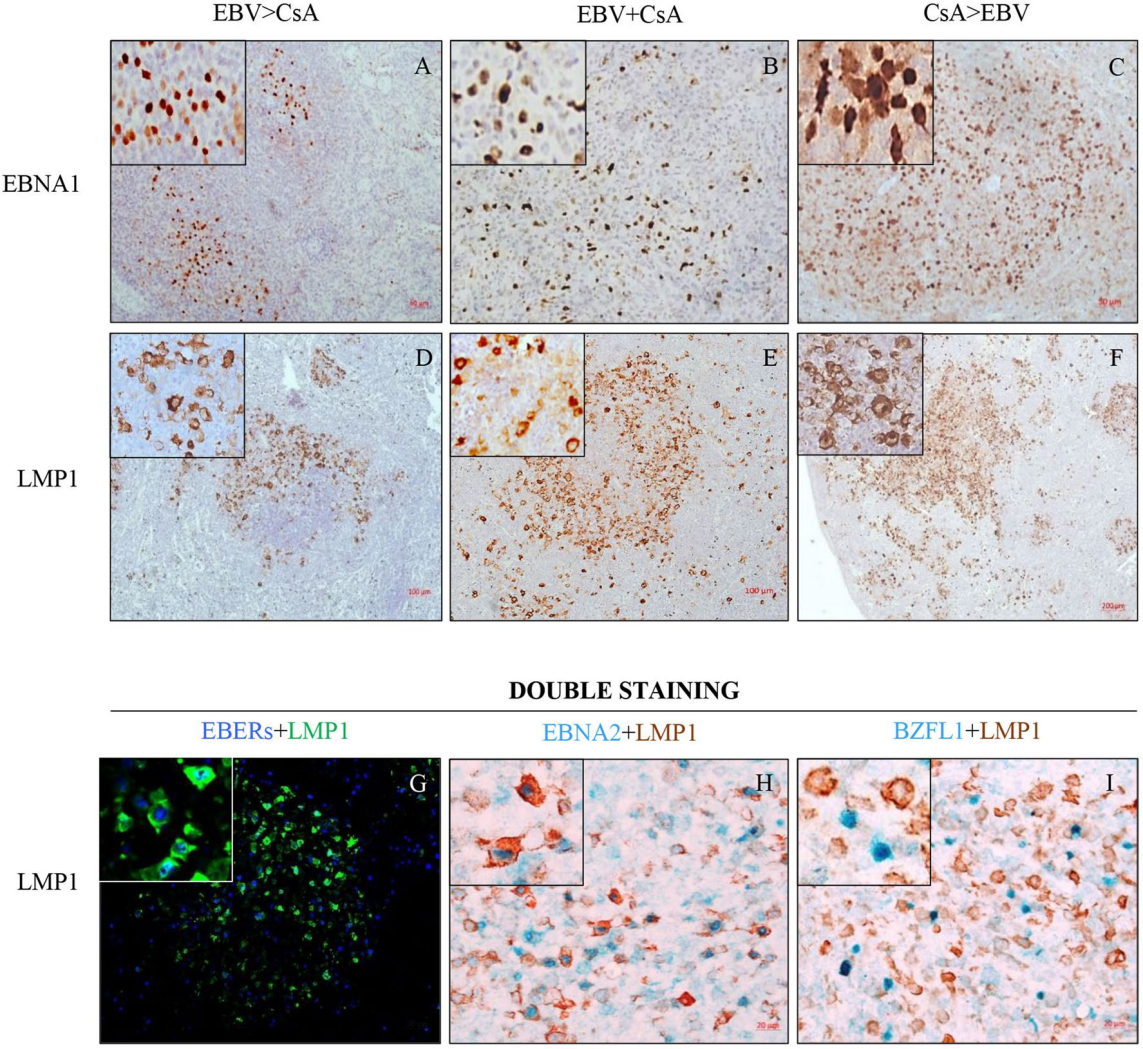
EBV infected cells in the spleen express different viral latent proteins. (**A**–**C**) EBNA1 and (**D–F**) LMP1 in the spleen from rabbits infected and immunosuppressed at different time points (EBV > CsA), (EBV + CsA) or (CsA > EBV). (**G**) Double immunofluorescence for EBERs (blue) and LMP1 (green) indicated that a large proportion of EBER^+^ cells also expressed LMP1. (**H**) Similarly, most of the EBNA2^+^ cells (blue) were also LMP1^+^ (brown). (I) By contrast, BZLF1^+^ cells (blue) did not co-express LMP1 (brown).

Immunohistochemistry was performed to determine the expression of various latent and lytic proteins in formalin-fixed, paraffin embedded spleen sections. Although both immunocompetent and immunosuppressed animals infected with EBV expressed various viral latent proteins (EBNA1, EBNA2 and LMP1), the degree of expression of these proteins differed between the groups. The spleens from immunosuppressed animals showed extensive and varied expression of EBNA1 (Fig. 3A–C), LMP1 (Fig. 3D–F), and to a lesser extent, the lytic protein, BZLF1 (sFig. 3). Among the three immunosuppressed groups, CsA > EBV group showed the most extensive and widespread EBV infection, and viral gene expression (Fig. 3C and F). We also observed multinucleated cells with marked increase in their size resembling Reed-Sternberg like cells in the spleen with extensive infections (sFig. 4). Notably, the expression of EBV latent proteins was most prominent in sites that resembled follicles of secondary lymphoid organs (Fig. 3D–F). We performed double-staining for latent and lytic proteins to determine whether these two programs co-expressed in EBV infected cells. The staining revealed that most, but not all, EBER^+^ cells expressed LMP1 in the spleen of immunocompromised animals (Fig. 3G and sFig. 4). Similarly, majority of the EBNA2^+^ cells also expressed LMP1 (Fig. 3H and sFig. 5). By contrast, double staining for BZLF1 and LMP1 revealed that, BZLF1^+^ cells were LMP1 negative (Fig. 3I and sFigs. 6 and 7). Thus, the lytic marker BZLF1 and latent marker LMP1 do not appear to be co-expressed at the same time in the same cell. Taken together, these double staining experiments indicate that in primary EBV infection in vivo, EBV infected cells at different patterns of viral gene expression can be seen in the same tissue (Fig. 3G–I, sFigs. 3–7).

We also noted that EBV infected cells were frequently present in the follicles. However, their distribution within the follicles varied considerably. In some cases, EBV infected cells heavily infiltrated the marginal/mantle zone (Fig. 3D–E), whilst in others, they were evenly distributed throughout the follicle (Fig. 3C). In cases where there was extensive infection, as in CsA > EBV group, multiple EBV positive follicles could be seen fused into one large network of infected cells, destroying the normal splenic follicular structure (Fig. 3F).

### EBV infected cells in the spleen proliferate in the follicles

Since EBV infected cells were frequently observed in the follicles of the spleen, we wanted to determine if virus-infected cells were proliferating in lymphoid follicles.

**Figure 4:**
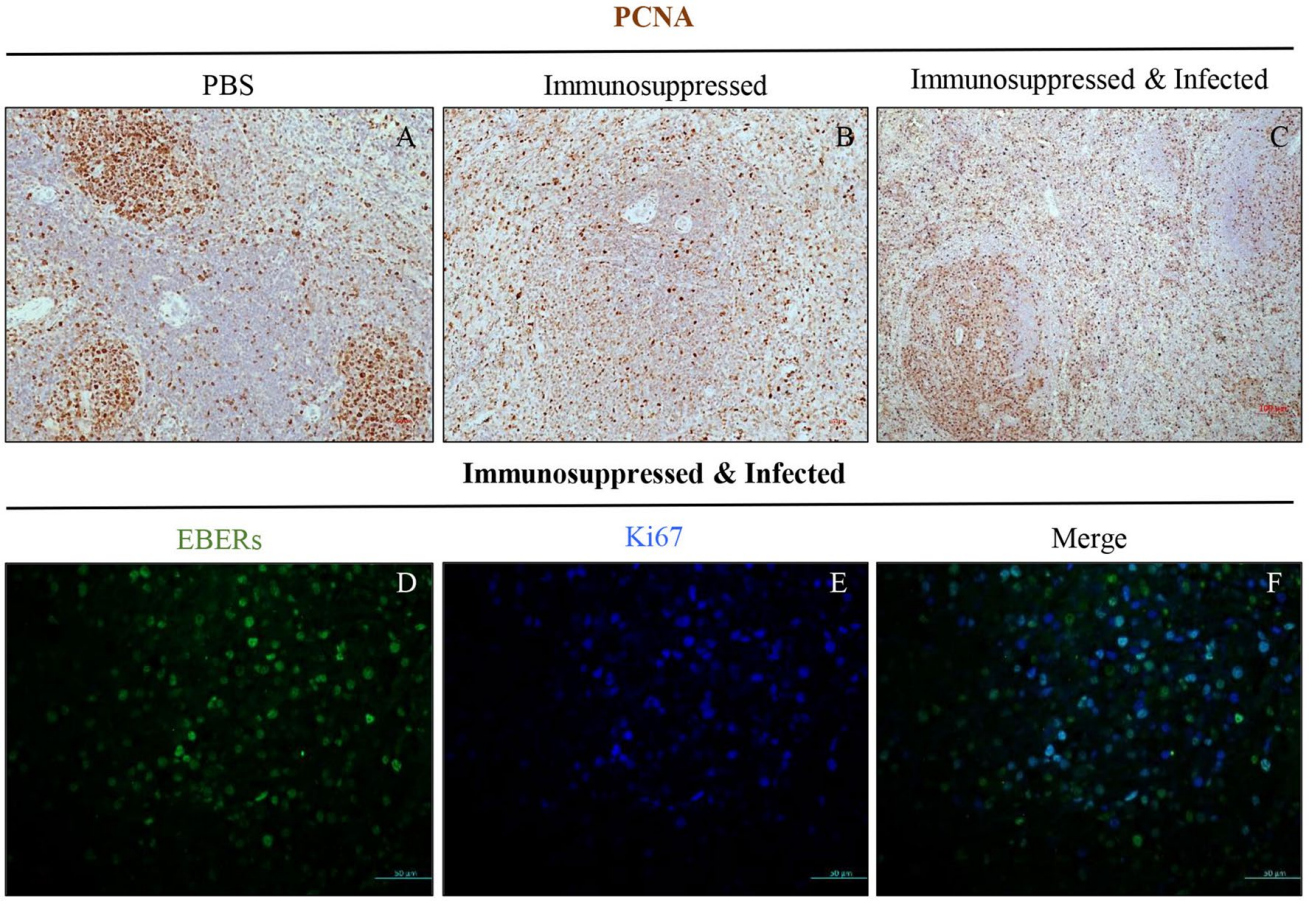
Infected cells proliferate in the lymphoid follicle. Representative spleen sections stained for PCNA, from: (**A**) Non-infected PBS control animal, (**B**) non-infected immunosuppressed control animal, and (**C**) EBV infected and immunosuppressed animal. Double immunofluorescence staining of spleen sections for: (**D**) EBERs (green), (**E**) Ki67 (blue) and (**F**) merged image shows that some of the EBER^+^ cells are also Ki67^+^.

In healthy non-infected spleen, prominent and strong PCNA/Ki67-positive cells were located in the germinal center (GC) of follicles (Fig. 4A). Tightly connected PCNA^+^ cells were organized into well defined GC structures. By contrast, in non-infected, immunosuppressed controls (CsA only), the PCNA^+^ cells were scattered in both the follicular and interfollicular regions (Fig. 4B). In the EBV infected and immunosuppressed animals, however, PCNA stained most of the cells in the follicles, and many in the interfollicular regions (Fig. 4C). It is noteworthy that we did not observe typical germinal center-like structures in immunosuppressed animals, whether infected or non-infected.

**Figure 5:**
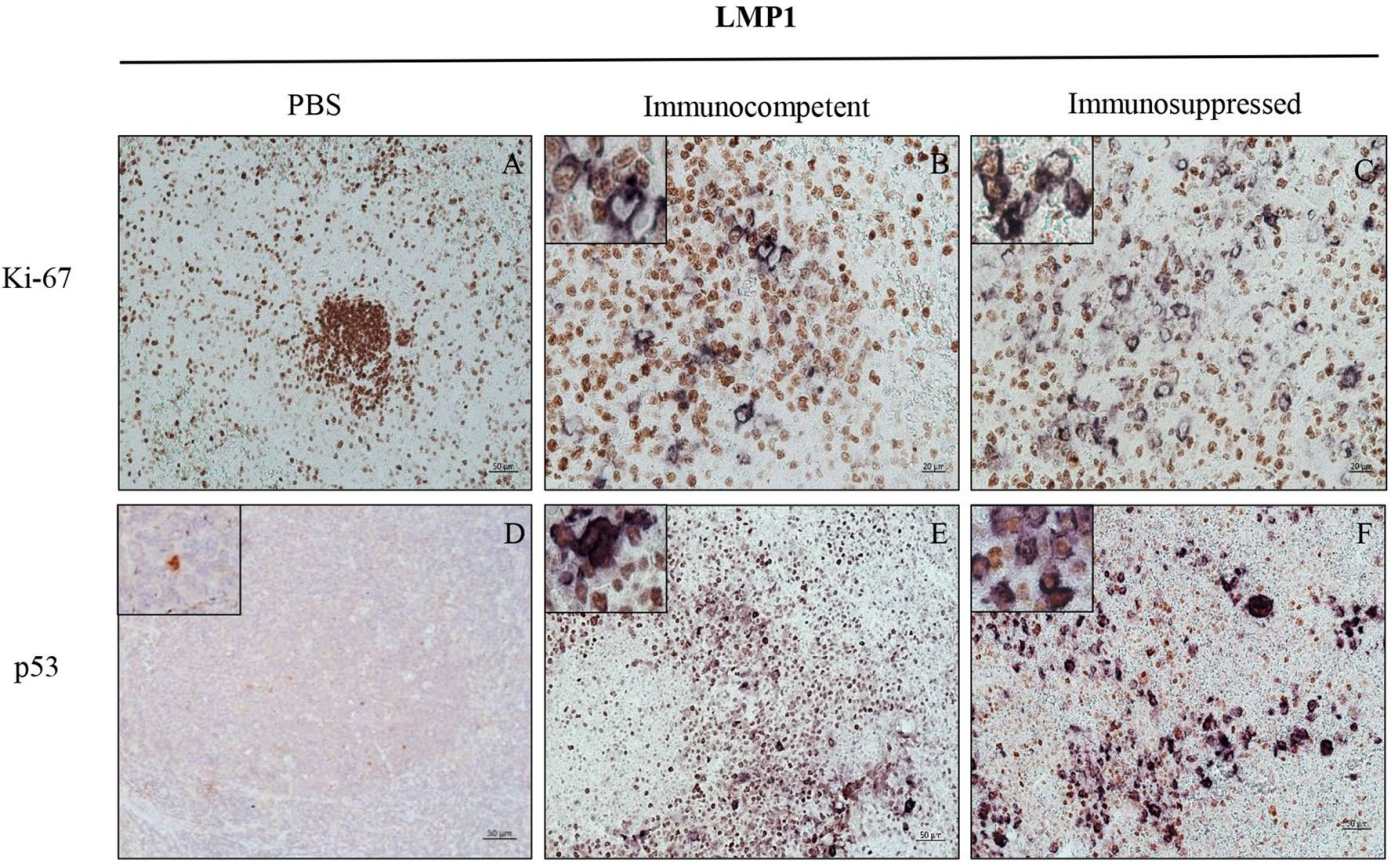
LMP1^+^ cells do not proliferate, but accumulate p53. Double staining for LMP1 (purple) and Ki67 (brown) in spleen, in: (**A**) non-infected PBS control, (**B**) infected immunocompetent animal, and (**C**) infected immunosuppressed animal. Double staining for p53 (brown) and LMP1 (purple), in: (**D**) non-infected PBS control, (**E**) infected immunocompetent animal, and (**F**) infected immunosuppressed animal.

While EBERs are the only EBV transcripts that are typically expressed in latency 0 of EBV infection, LMP-1 is expressed in latency II/III. To determine which EBV infected cells were proliferating in the spleen of rabbits, we performed double staining for EBERs and Ki-67, and LMP1 and Ki-67. The results indicated that EBER-positive cells were generally small and Ki67 positive (Fig. 4D–F). Surprisingly, the large LMP1-positive cells were Ki67 negative, indicating that these cells were not proliferating (Fig. 5B–C). Moreover, LMP1-positive cells were also found to be p53 positive (Fig. 5E–F). As expected, in the PBS non-infected control animals, no expression of LMP1 was observed. Although, Ki67 proliferating cells were abundantly present in the spleen from the PBS control animals (Fig. 5A), very little expression of p53 was observed (Fig. 5D).

### EBV infected cells do not express the GC marker BCL6

**Figure 6:**
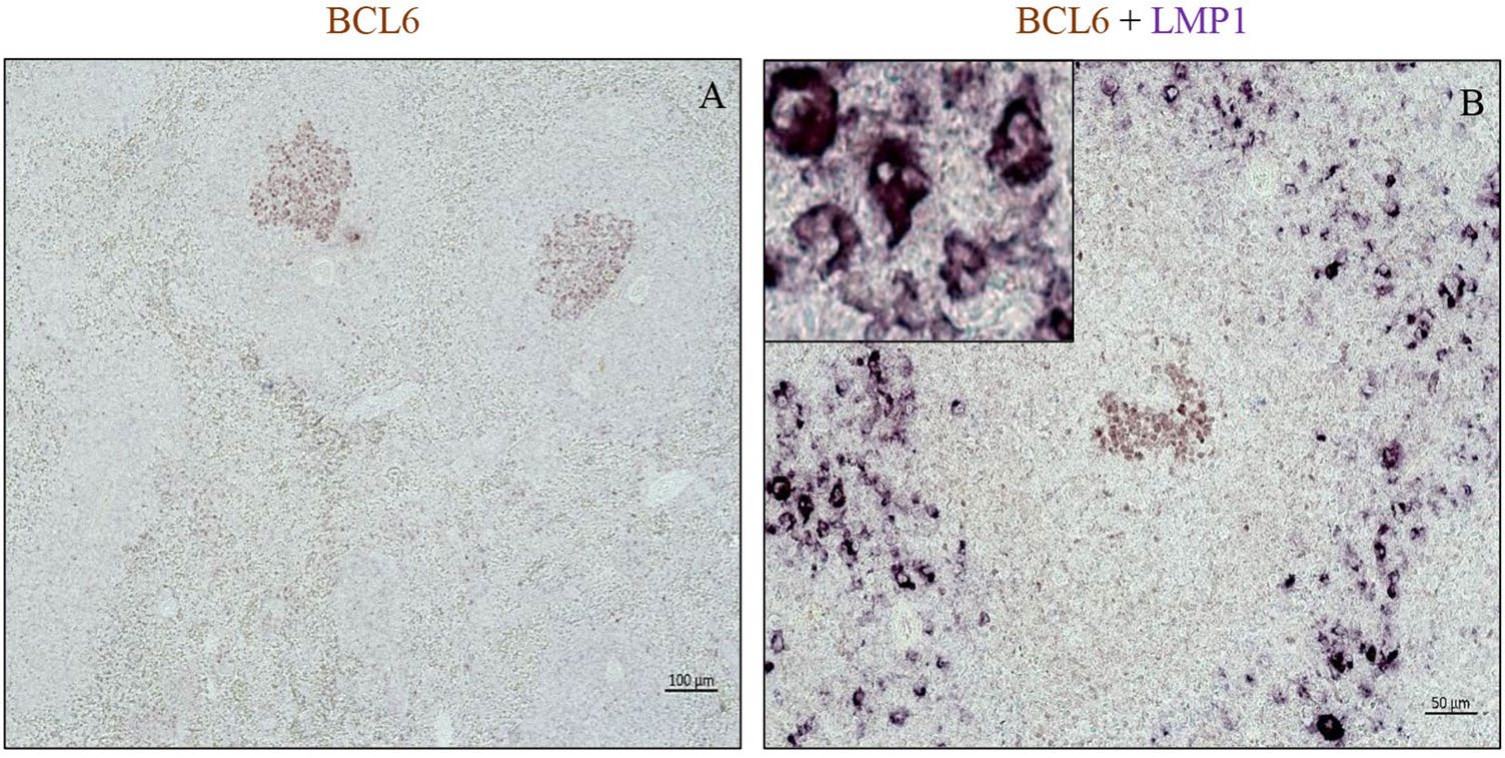
EBV infected cells in the GC do not express GC marker. Double staining for BCL6 (brown) and LMP1 (purple) in spleen, in: (**A**) non-infected PBS control animal, and (**B**) infected immunocompetent animal (**B**).

To determine if EBV infected cells were germinal center cells, we performed double staining for LMP1 and BCL6, a marker of germinal center (GC) cells. No co-expression of these markers was observed (Fig. 6B), indicating that EBV-LMP1^+^ cells are not GC cells. Indeed, LMP1^+^ and BCL6^+^ cells were spatially distributed in different regions of the follicle (Fig. 6B). We also observed that BCL6^+^ cells were less abundant in the spleen of EBV infected animals compared to non-infected PBS control (Fig. 6A).

## Discussion

Epstein-Barr virus (EBV) is a common herpesvirus asymptomatically carried by over 90% of the human population. In the vast majority of infected individuals, the virus persists in B-cells without causing any symptomatic disease^30, 31^. However, in certain circumstances, such as in immunocompromised states, this virus can lead to life threatening diseases including malignancies^32–34^. Unfortunately, several fundamental aspects of the biology of EBV and its association with human diseases remain poorly understood. The lack of a suitable animal model for EBV infection has contributed to this limitation. Recent evidence suggests that rabbits can be susceptible to EBV infection, and can be used to study long term infection in vivo^24, 25, 35^. In these animals, the virus persist asymptomatically for months. These features are similar to latent EBV infection seen in healthy human carriers^9, 23^. When infected rabbits are immunosuppressed, peripheral EBV load increases from undetectable to several thousand copies^24^. Additionally, EBV infected rabbits have been shown to mount a strong humoral immune response against the virus^36^. Using this rabbit model, we report, for the first time, the early events during primary EBV infection in vivo.

Intravenous introduction of EBV resulted in all animals contracting the virus. In contrast to the immunocompetent animals, the spleen from infected immunosuppressed group, showed marked splenomegaly and visible inflammatory nodules^24, 35^. On histology, the enlarged spleens showed evidence of extensive infection and destruction of the splenic architecture. It is likely that the weakened immune system allowed infected cells to undergo rapid proliferation. As a result, large number of EBV infected cells contributed to marked increase in the size of the spleen. Furthermore, of the three immunocompromised groups which were infected, the animals that were immunosuppressed for one week followed by EBV infection (CsA > EBV) had the most extensive infection, reminiscent of what has been observed in patients with congenital immunodeficiencies, such as X-linked lymphoproliferative disease (XLP)^37^. These patients typically develop EBV-associated lymphoproliferative diseases on primary infection and die early in childhood^38, 39^. In addition to the extensive infection, EBV infected cells in immunocompromised animals, were transcriptionally active and expressed a range of viral proteins. In the EBV > CsA group, the host immune system was not suppressed initially for 1 week. This allowed the immune system to control the viral infection. Upon starting CsA treatment at week 2, the virus faced relatively less efficient immune response, which facilitated the virus spreading to major organs such as spleen and liver. However, in CsA > EBV group, the suppression of immune system 1 week prior to infection, provided the opportunity for the rapid proliferation of the infected cells as soon as the virus gained entry into the host. During the primary EBV infection and persistence, hierarchical immune response is driven by specific latent and lytic gene expression^40^. Despite the concomitant presence of infected cells expressing latent and lytic viral markers in the same follicle, we did not observe co-expression of lytic and latent proteins at the individual cell level, at least using immunohistochemistry. This observation is supported by reports that indicate that there is little or no overlap in the coexpression of LMP1 and BZLF1 in in vitro infected cells^41^. One plausible explanation is that LMP1 could be inhibiting viral lytic cycle^42^. Furthermore, some studies have reported that BZLF1 positive cells in tumors were in fact negative for EBV latent products, such as EBERs^43, 44^. Taken together, these observations suggests that during early infection in vivo, at any given time point, the cells are either in latent or lytic cycle, but not both.

Another interesting observation was that, latently infected cells were not synchronous, and cells in all the latency programs (0–3) were observed. This is in contrast to long-term persistence infection in healthy seropositive individuals where the virus persists in memory B-cells in latency 0^30^. It is noteworthy that some infected cells were distinctly large, and frequently multinuclear. These Reed-Sternberg (RS) like cells were typically LMP1 positive. Previous in vitro studies have reported that LMP1 could mediate the development of these multi-nucleated cells^8, 45, 46^. In heavily infected cases, the loss of well-defined B-cell areas in the spleen, indicates that the increased burden of EBV infection can result in the breakdown of the well-defined follicular architecture. As a result, scattered, rather than clustered, B cells characterized the nearly destroyed follicle. Compared to their non-infected counterparts, infected B cells showed weaker CD79a, but higher IgM expression. This suggests that, EBV infection may alter the expression of some important B cell markers such as CD79a and IgM^47–50^. The infected cells also seemed to reside primarily in the follicles, since splenic follicles are crucial site for B cell development. The majority of the infected cells inside the follicles were B cells. The infected cells expressed various B cell markers, including IgM, CD79a, CD20 and CD21. However, the intensity of staining for these markers varied. We hypothesize that the infected cells were initially restricted to the follicles, but as the infection spreads in situ, the normal structure of the follicles is destroyed. Although infected cells were abundantly present inside the follicles, they did not express the GC marker BCL6. This data is consistent with a previous study which reported that EBV infected cells can expand in the GCs, but do not necessarily participate in the GC reaction^51^. It is possible that EBV infected cells increase in number without undergoing somatic hypermutation^26^.

Another key observation was that most of the small EBER^+^ cells were found to be proliferating (Ki-67^+^), whereas the large LMP1^+^ cells were non-proliferating and p53^+^. This was somewhat surprising, as LMP1 is known to be an essential viral protein involved in cell proliferation and transformation. In epithelial cells, it has been reported that the expression of LMP1 can result in p53 accumulation, but p53-mediated apoptosis is inhibited by LMP1 induced expression of anti-apoptotic factors such as BCL-2 and A20^52–54^.

Taken together, we propose that in primary EBV infection in vivo, the virus targets naïve B-cells in secondary lymphoid organs, such as the spleen, become blast cells and undergo proliferation. These infected cells reach the follicles and occupy the marginal zone. Once in the marginal zone, some infected cells undergo productive cycle, shedding new virus particles, whilst others go through latent cycle expressing various latent genes. Among the cells expressing latent proteins, a portion of these cells undergo proliferation. While the infected cells are in the marginal zone, the GC remains intact and shows increased expression of BCL6 and Ki67. As the infection begins to spread through the mantle zone and further towards the GC, it begins to disrupt the structure of the GC and the size of the GC diminishes. These infected cells show increased expression of p53, which is not expressed in non-infected cells. As the number of EBV infected cells in the follicle increases, the number of BCL6^+^ GC cells gradually decline. Eventually, the entire follicle becomes filled with EBV infected cells with no intact GC. The EBV infected follicles expand, losing their well demarcated cellular zones. Multiple EBV infected follicles eventually fuse, resulting in the destruction of the typical follicular architecture. Based on our observations, we believe that follicles play an important role in EBV infection and proliferation, but GCs do not appear to be involved. However, further research is needed to verify this hypothesis. The rabbit model of EBV infection described here, will certainly help in further delineating the steps involved in primary EBV infection and the development of EBV induced malignancies. Additionally, this model may prove to be essential for vaccine and therapeutic developments against EBV.

## Materials and methods

### Animals

Healthy New Zealand white rabbits (n = 30), aged approximately 4–8 weeks and weighing 700-1000 g were purchased from a local vendor and housed in the animal facility of College of Medicine and Health Sciences, UAE University. All animals were tagged, weighed, and kept in the animal house for 2 weeks to acclimatize to their new environment before performing any experiments. All experiments were conducted in accordance with guidelines provided by the UAE University Research Ethics Review Board (RERB) and approved by the Institutional (UAE University) Animal Research Ethics Committee (A-REC) (approval #A15-15: ERA 2018–5718). The study was carried out in compliance with the ARRIVE guidelines.

### Preparation of EBV inoculum for infection

EBV producing B95-8 cells were cultured at 37 °C in RPMI (GIBCO) supplemented with 10% Fetal Bovine Serum (FBS) (GIBCO), 50 µg/ml gentamycin (Hyclone), 1% antibiotic and antimycotic solution (Santa Cruz) and 1X glutamine (GIBCO), as previously described^24^. Cells were grown to a density of approximately 6.5 × 10^6^cells/ml and then incubated at 30ºC for 24 h to promote viral production. The culture supernatant was centrifuged to remove cell debris and filtered using a 0.2 µm filter. Freshly filtered culture supernatant containing the virus was then used for intravenous inoculation of rabbits. Quantitative real-time PCR (qRT-PCR) was used to determine EBV copy number in the inoculum, using Namalwa cell line DNA as standards^55^.

### EBV infection and immunosuppression of rabbits

Animals were infected with a total of 1 ml of fresh filtered culture supernatant containing 1 × 10^7^ EBV copies, by intravenous injection into the marginal ear vein.

The animals were divided into 4 groups:

- ***Group 1: EBV infected—immunocompetent*** (n = 5): All animals in this group received intravenous injection of 1 × 10^7^ copies of EBV. All animals were sacrificed 2 weeks post EBV infection.
- ***Group 2: EBV infected—immunosuppressed*** (n = 18): All animals received the same dose of EBV as in group 1. However, these animals were immunosuppressed by daily subcutaneous injection of 20 mg/Kg of cyclosporin A (CsA) (Sandimmume, Novartis) until the day of sacrifice, starting at:
  A. 1 week before EBV infection (CsA > EBV) (n = 6)
  B. Same time as EBV infection (CsA + EBV) (n = 6)
  C. 1 week post EBV infection (EBV > CsA) (n = 6)
- All animals were sacrificed 2 weeks post EBV infection/CsA administration.
- ***Group 3: Non-infected—immunocompetent control*** (n = 4): All animals in this group received sterile isotonic saline (PBS) instead of EBV. All animals were sacrificed 2 weeks post PBS administration.
- ***Group 4: Non-infected—immunosuppressed control ***(n = 3): All animals in this group received PBS followed by daily injections of CsA. All animals were sacrificed 2 weeks post CsA administration.

All the animals were monitored on a daily basis by a veterinarian, for any signs of diarrhea, weight loss or CsA toxicity. Any animal showing signs of disease or distress were sacrificed in accordance with the ethical guidelines. On the day of sacrifice, animals were euthanized, autopsied and spleen and whole blood were harvested. Peripheral blood mononuclear cells (PBMCs) and plasma were isolated from whole blood using Histopaque 1083 (Sigma). The PBMCs and plasma were then store at − 80 °C and − 40 °C, respectively, till further analysis.

### EBER-in situ hybridization for the detection of EBV

EBV in the spleen was detected using in situ hybridization targeting the abundantly expressed EBV-encoded RNAs (EBERs), as previously described^43, 44^. Briefly, EBER-ISH was performed on 5-μm sections of formalin-fixed, paraffin-embedded spleen tissue using a mixture of digoxigenin-labeled EBER-1 and EBER-2 probes with overnight hybridization. Hybridized probes were detected using anti-digoxigenin mouse monoclonal antibody at a dilution of 1/2500, and the ABC-peroxidase detection kit (ThermoFischer Scientific, USA). Diaminobenzidine tetrahydrochloride (DAB) (Sigma, UK) was used as the chromogen. With each batch of tissue sections, a positive control (a known EBV-positive rabbit spleen) and a negative control (using digoxigenin-labeled non-complimentary EBER probes) were included^24^. Sections were counterstained with hematoxylin, and slides were mounted.

### Determination of EBV viral load by qPCR

DNA was extracted from rabbit samples using either the standard phenol–chloroform method, or DNA extraction kit (Qiagen). EBV copy number in the plasma, PBMCs and spleen was quantified using qPCR targeting EBV BamH1 W fragment, and Namalwa cell line DNA as standards^56^. For each reaction, 50 ng of template DNA was used in a total volume of 20 μl using ABS TaqMan Universal Master Mix along with TaqMan probe. All the samples were tested either in duplicates or triplicates and independently repeated 3 times in a 40 cycle reaction using Applied Biosystem Quantstudio-7 (AB7500) real time PCR machine^24^.

### Immunohistochemistry for viral and cellular makers

Immunohistochemistry (IHC) was performed on 5-μm sections of formalin-fixed paraffin embedded rabbit spleen tissues. After deparaffinization with xylene, the sections were dehydrated in graded ethanol and endogenous peroxidase activity was blocked by incubating in 0.5% H_2_O_2_ in methanol for 20 min. Antigen retrieval was performed by incubating the sections in boiling 5 mM citrate buffer at pH6 for 10 min. Subsequently, sections were blocked with 5% BSA for 30 min at room temperature. Sections were then incubated in primary antibody for overnight, followed by secondary antibody for 30 min in blocking buffer (Table 1). With each batch of tissue sections, a positive control (a known EBV-positive rabbit spleen) and a negative control (a known EBV-negative rabbit spleen) were included^24^. The signal was developed using ABC-peroxidase/DAB detection system, and sections were counterstained with hematoxylin.

**Table 1:** Antibodies used in immunohistochemistry.

Clone	Target	Source	Specificity	Dilution	Company
CS1-4	LMP-1	Mouse	Viral	1:1000	Abcam
D-24G	LMP-1	Rabbit	Viral	1:200	Thermo
D810H	EBNA-1	Mouse	Viral	1:20	Thermo
PE2	EBNA-1	Mouse	Viral	1:100	Abcam
HM-57	CD79a	Mouse	Human/Rabbit	1:200	Abcam
mu chain	IgM	Goat	Rabbit	1:500	Abcam
CD3-12	CD3	Rat	Human/Rabbit	1:100	Abcam
EP3093	CD21	Rabbit	Human/Rabbit	1:300	Abcam
BCL6/1527	BCL6	Mouse	Human/Rabbit	1:500	Abcam
DO-1	P53	Mouse	Human/Rabbit	1:250	Santa Cruz
BZ1	BZLF1	Mouse	Viral	1:200	Santa Cruz
B56	Ki67	Mouse	Human/Rabbit	1:250	BD Biosciences
PC10	PCNA	Mouse	Human/Rabbit	1:2000	Abcam

### Immunofluorescence (IF) staining for viral and cellular markers

For IF staining, the procedure was similar to IHC, except that the endogenous peroxidase blocking step was omitted and fluorochrome-conjugated secondary antibodies were used. To reduce autofluorescence, sections were incubated in Vector TrueView quenching kit (Vector Lab, USA) for 2–5 min. Finally, sections were mounted in Fluoromount (Sigma) and examined using fluorescence microscope.

### Double-immunostaining for viral and cellular markers

Double-immunostaining was performed on 5-μm sections, following the IHC protocol. After developing the first antibody using ABC-peroxidase/DAB detection system, the sections were washed, followed by antigen retrieval for the 2^nd^ time. The sections were then blocked again using 5% BSA for 30 min, followed by overnight incubation in the second primary antibody. Secondary antibody conjugated with alkaline phosphatase (AP) was then added and the sections were incubated for 30 min. The sections were then blocked in alkaline phosphatase buffer pH 9.5 for 30 min, followed by NBT-BCIP substrate (Abcam). The slides are then gently washed, mounted and viewed under microscope. For IF double staining, a combination of primary antibodies were used (Table 1). Sections were incubated in 2 primary antibodies against 2 different targets (viral and/or cellular) overnight at room temperature. The following day, secondary antibodies labelled with different fluorochromes were used. Sections were quenched, mounted and examined using a fluorescence microscope.

### Statistical analysis

Statistical analysis was performed using Kruskal-Wallis test and Prism software version 9.1.2 (GraphPad). Data expressed here are as means ± standard error of the mean (SEM). *P* value ≤ 0.05 was considered statistically significant.

## Supplementary Information

Supplementary Information.

## Data Availability

The datasets used and/or analyzed during the current study are available from the corresponding author on reasonable request.
