# Supplementary material for: Uncovering early events in primary Epstein-Barr virus infection using a rabbit model
Source: Sci Rep. 2021 Oct 27;11:21220. doi: 10.1038/s41598-021-00668-x (PMC8551192; doi:10.1038/s41598-021-00668-x)

Supplementary Figures

sFig.1: Double staining: EBERs and CD19/CD20 (spleen)

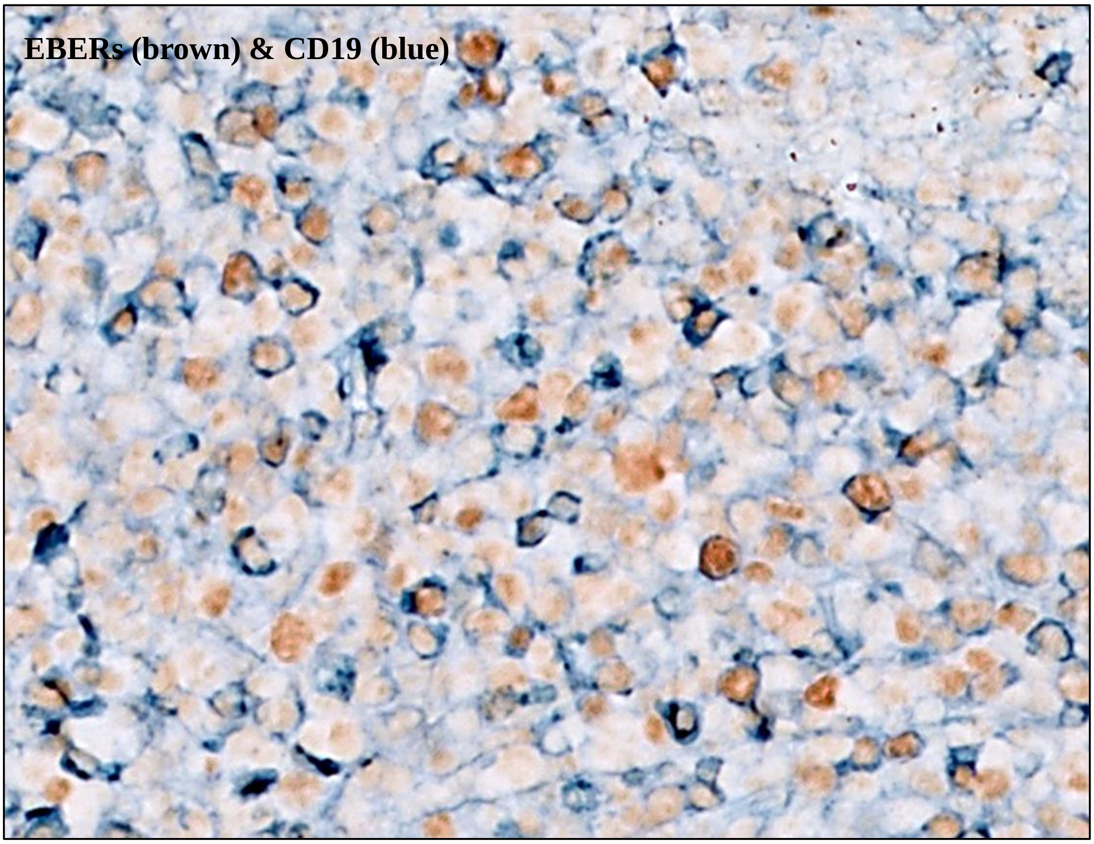

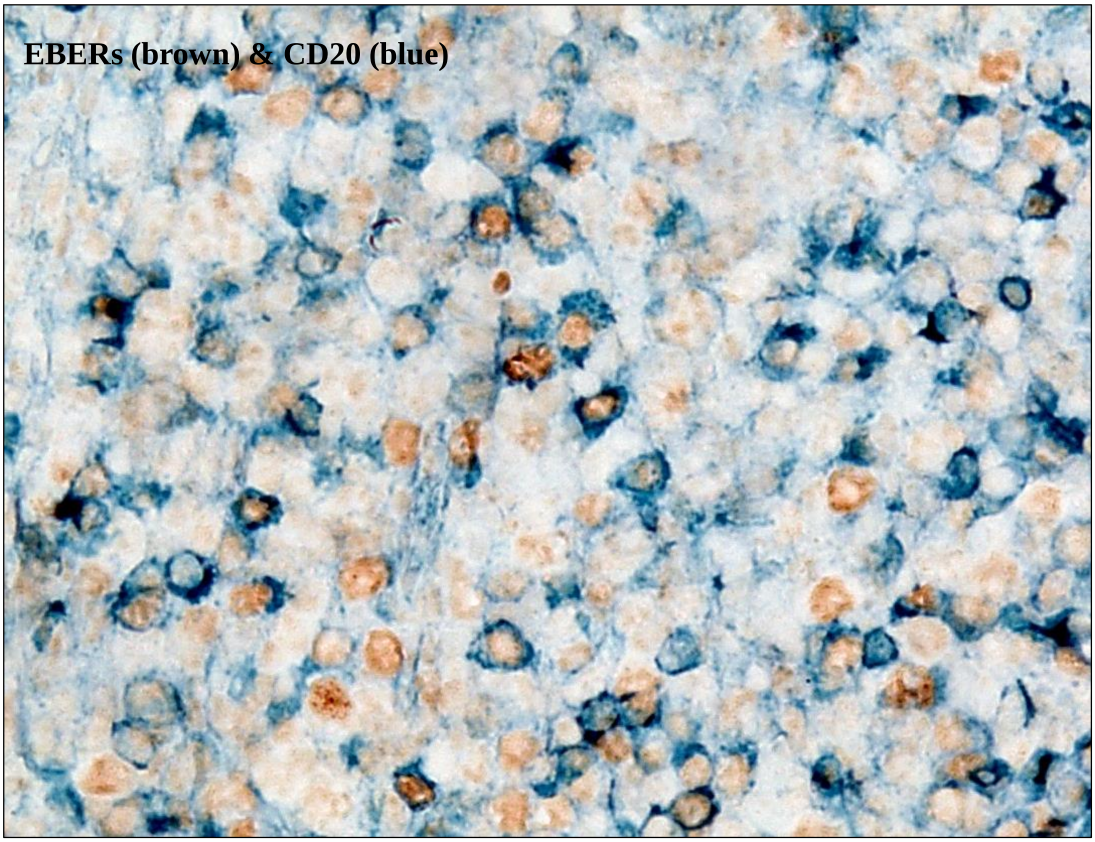

sFig.2: Immunohistochemistry double staining: EBERs & IgM (spleen)

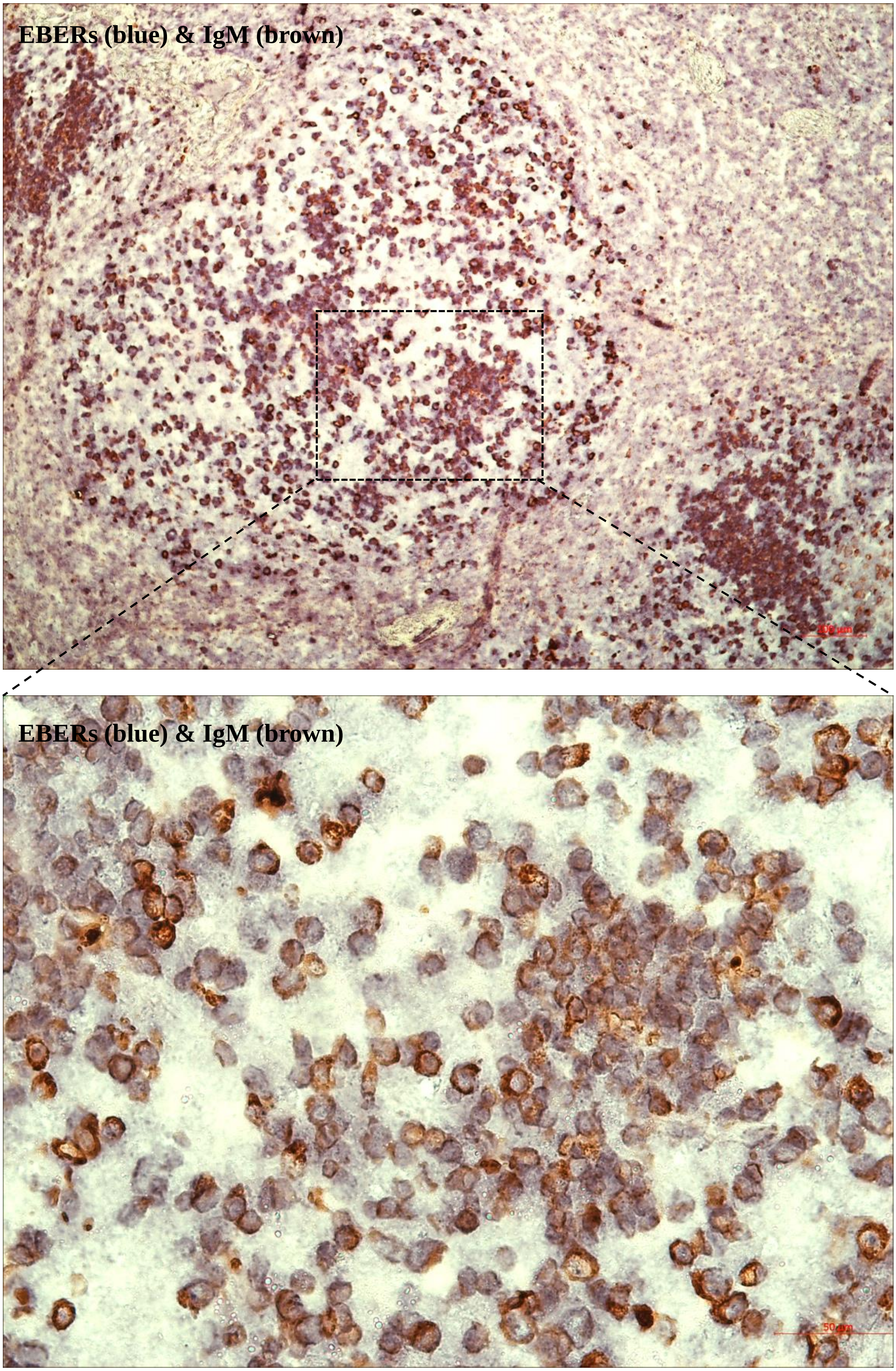

sFig.3: Immunohistochemistry staining: BZLF1 (spleen)

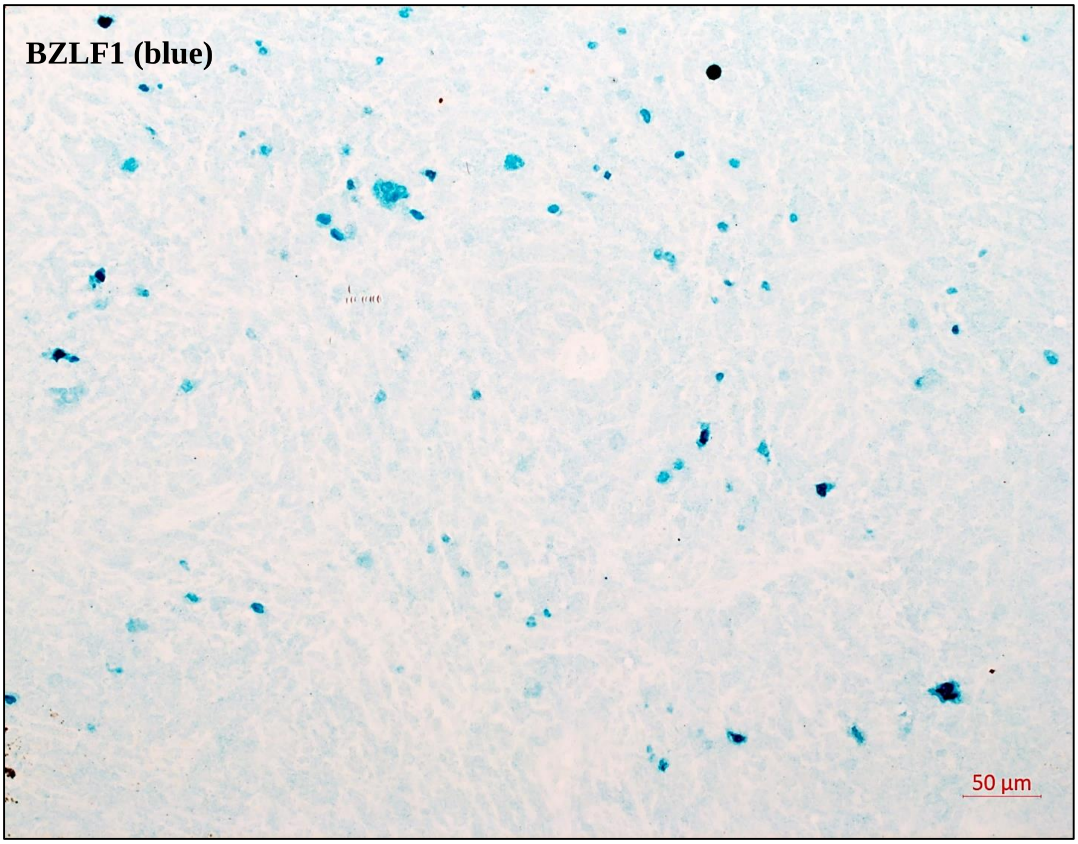

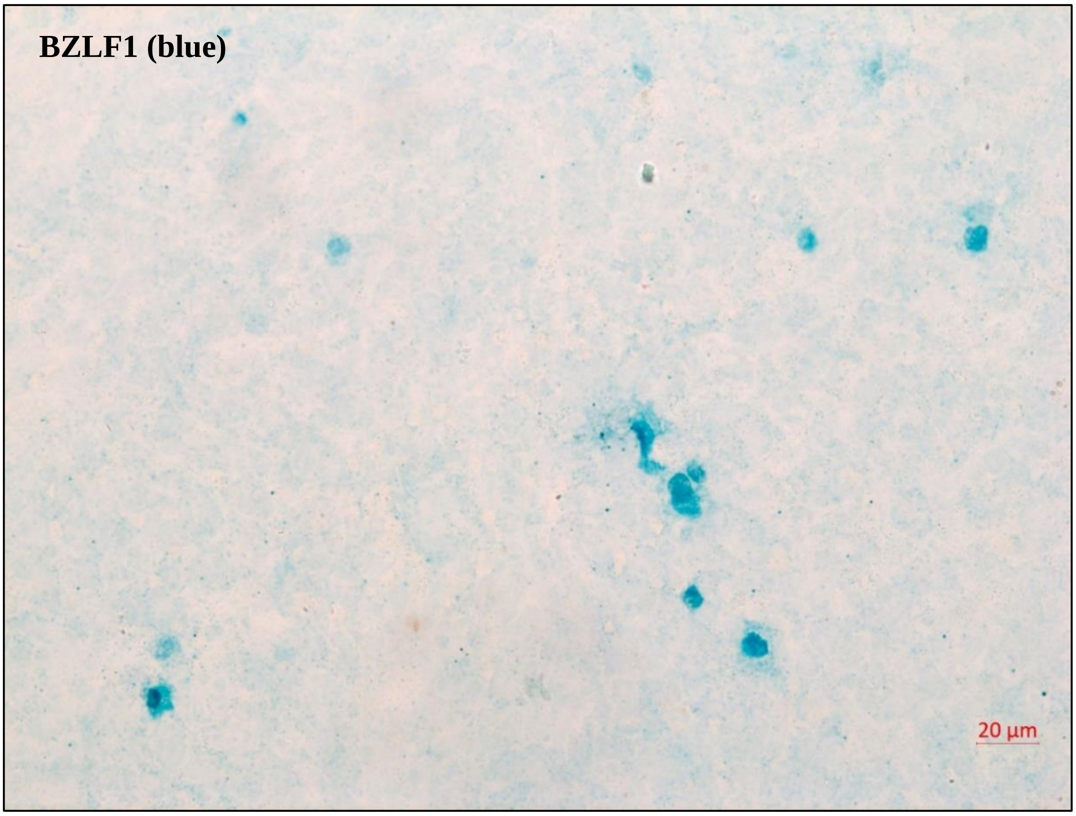

sFig.4: Immunohistochemistry double staining: LMP1 & EBNA2 (spleen)

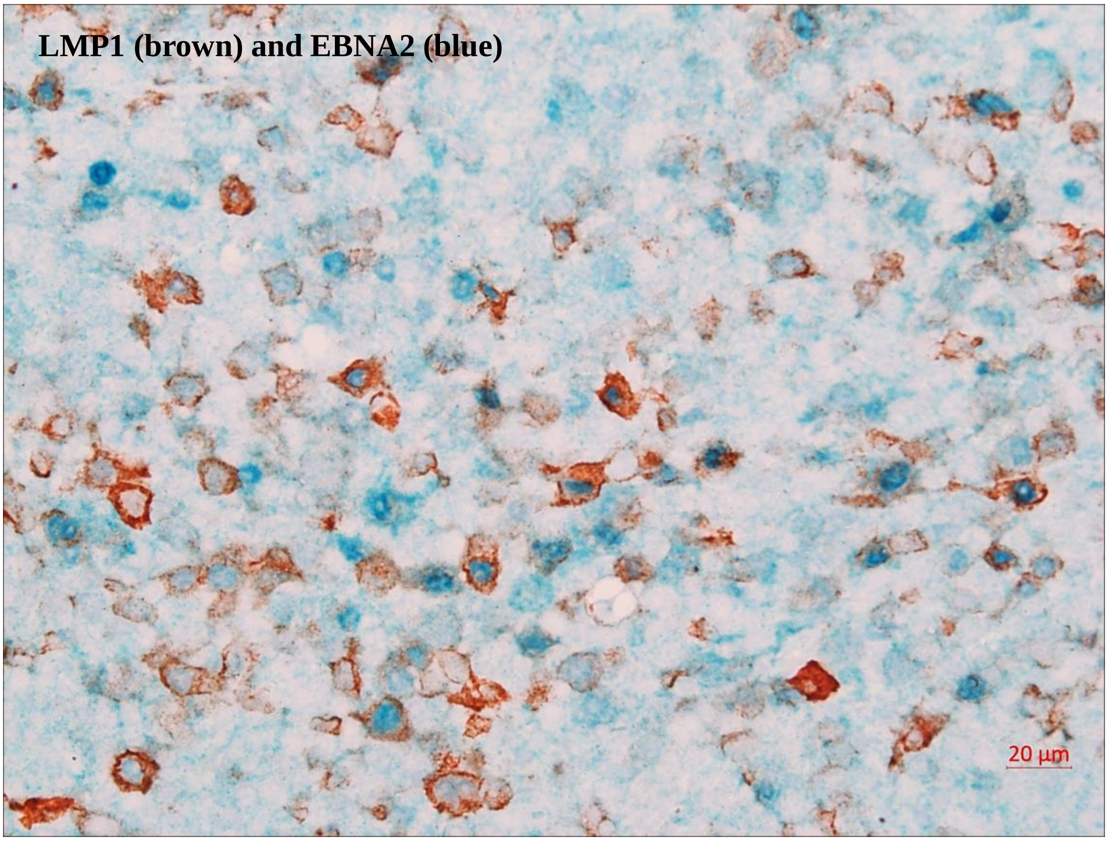

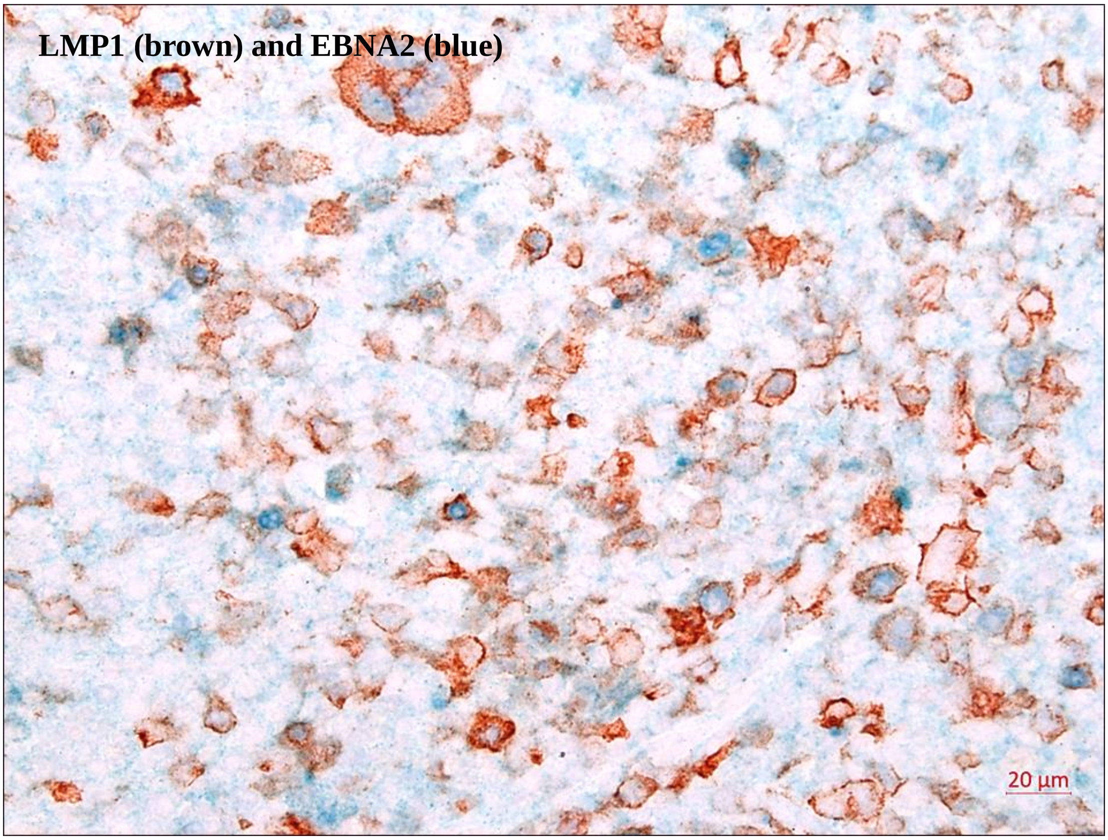

sFig.5: Immunofluorescence double staining: LMP1 and EBERs (spleen)

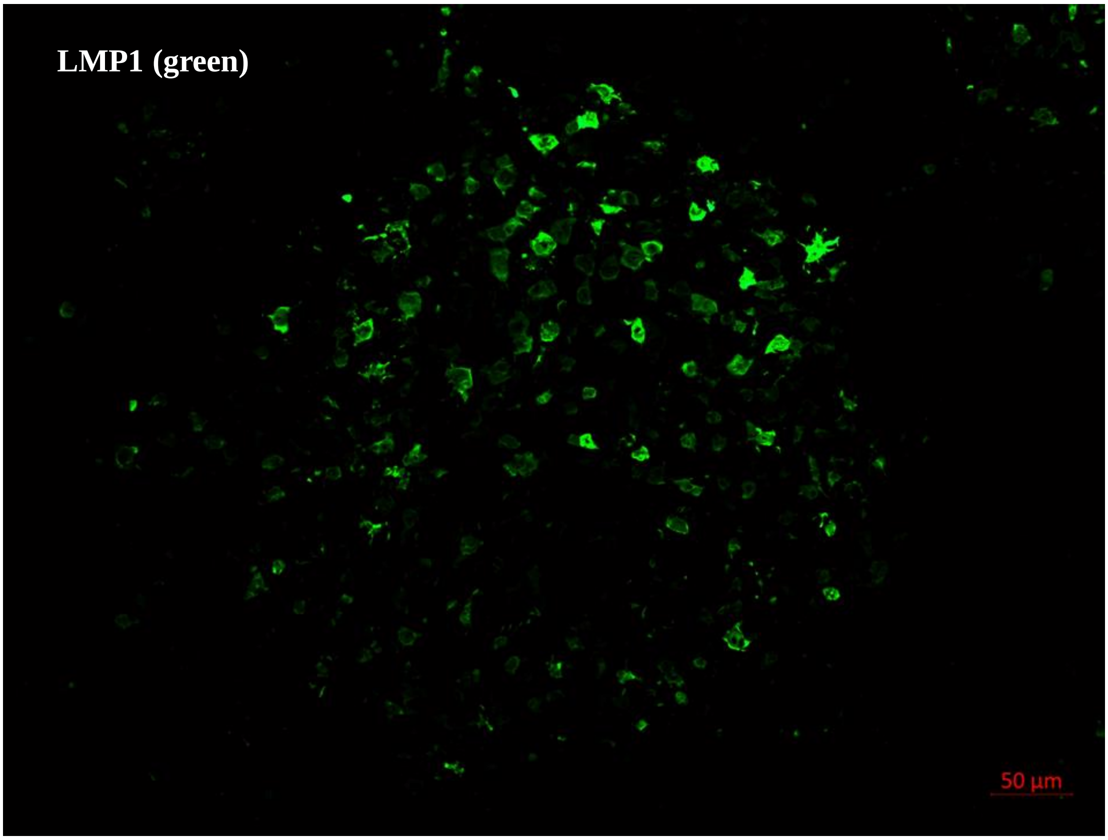

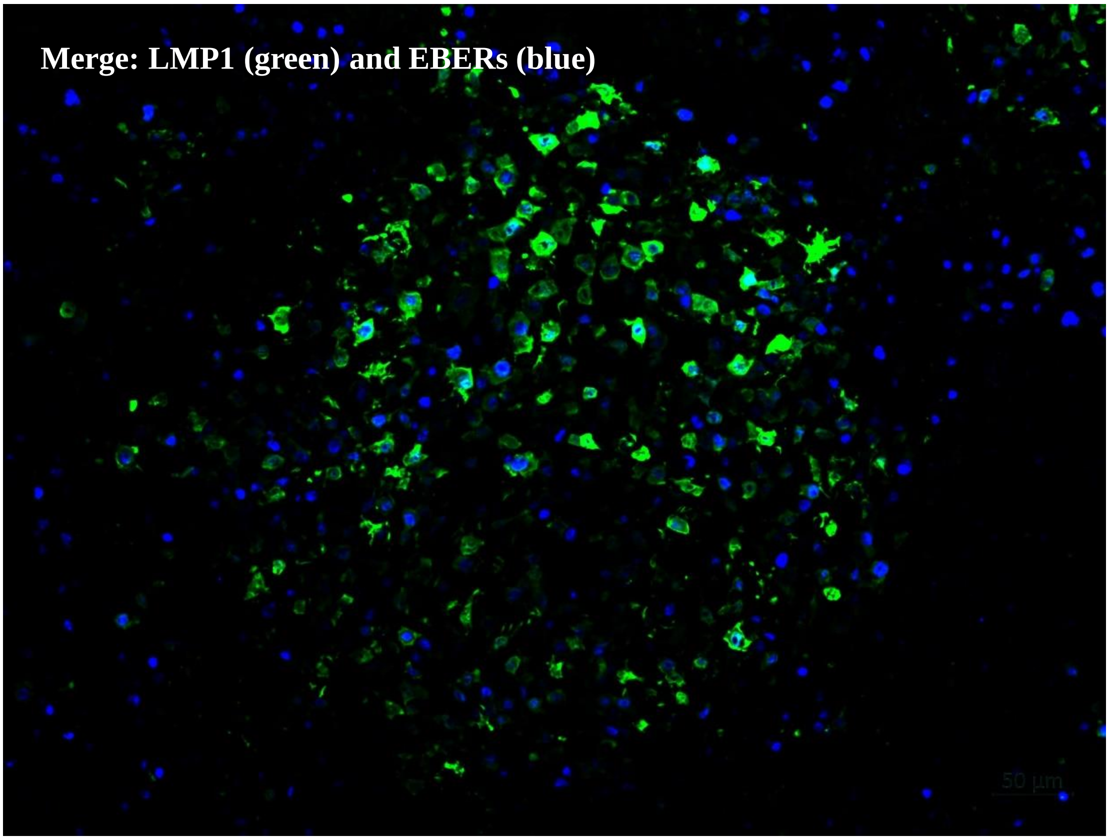

sFig.6: Immunohistochemistry double staining: LMP1 and BZLF1 (spleen)

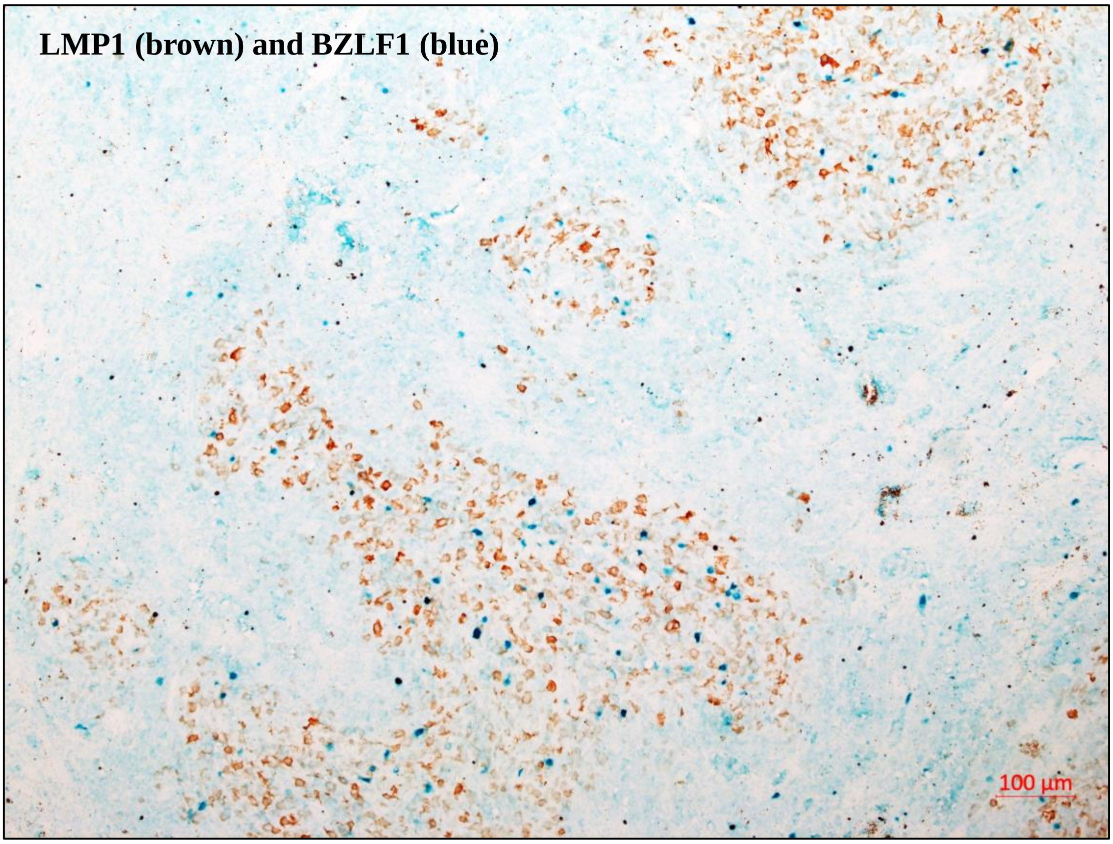

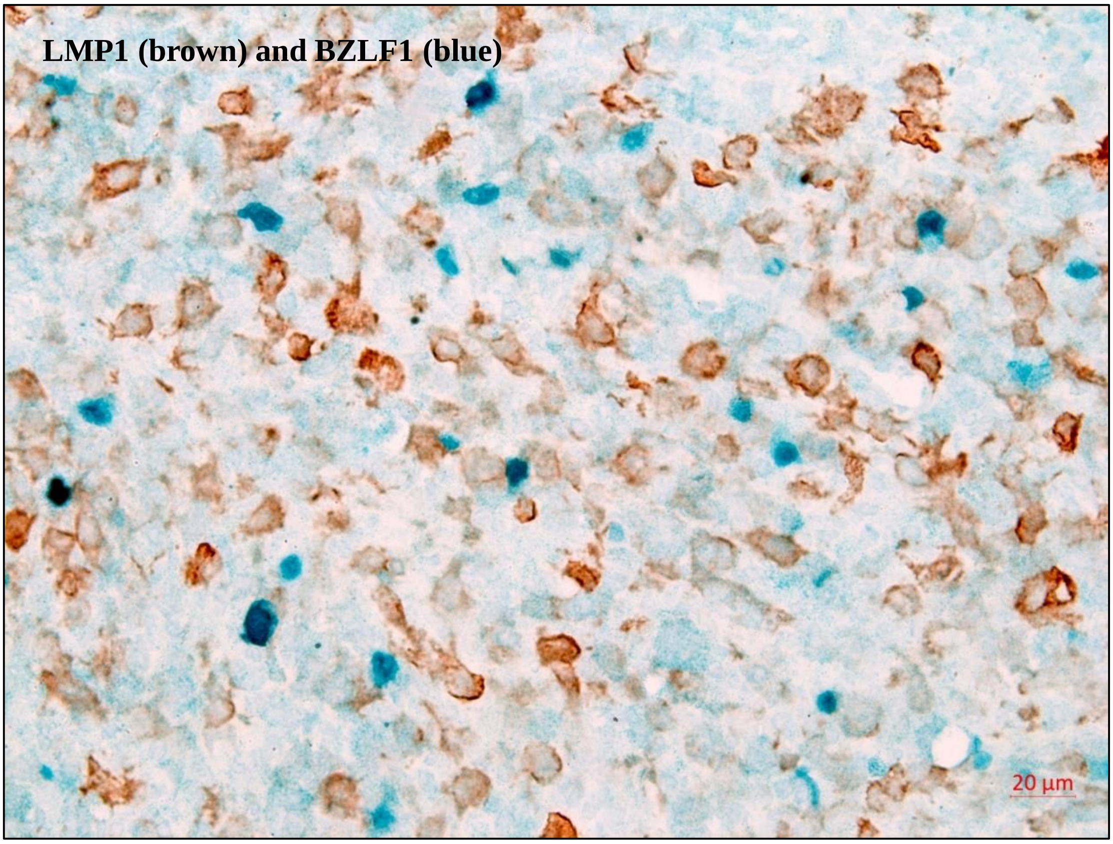

sFig.7: Immunohistochemistry double staining: LMP1 & BZLF1 (spleen)

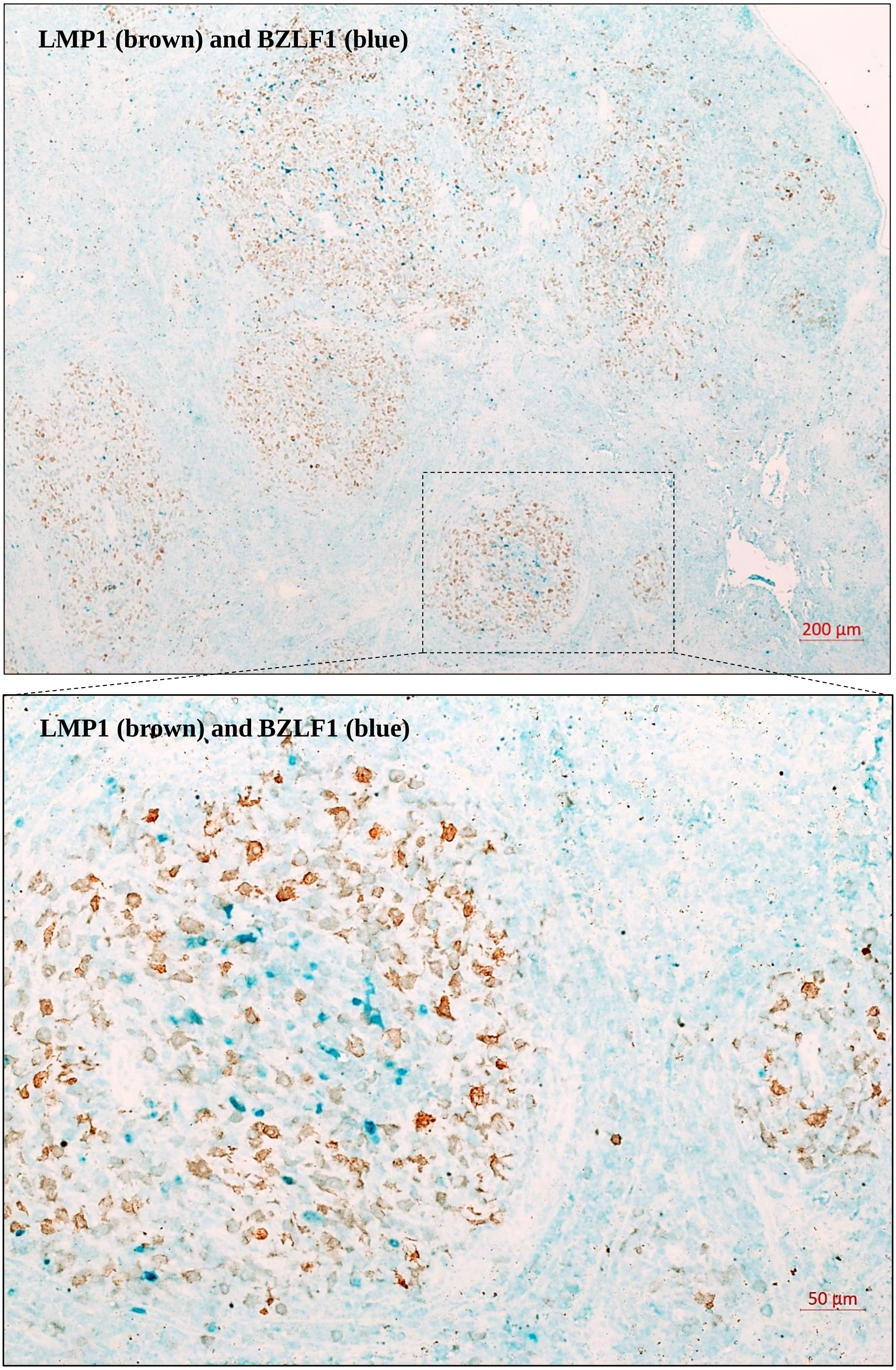

Supplement: Supplementary file 1 — Supplementary Information. [file 41598_2021_668_MOESM1_ESM.pdf]
